# Observation of Ras protein GTP hydrolysis and conformation change by freeze-trap serial X-ray crystallography

**DOI:** 10.1063/4.0001157

**Published:** 2025-10-27

**Authors:** Takashi Kawamura, Wakako Sakisaka, Yoshiteru Makino, Shigeyuki Matsumoto, Yoko Yoshikawa, Kazuya Hasegawa, Fumi Shima, Takashi Kumasaka

**Affiliations:** 1 Japan Synchrotron Radiation Research Institute, Sayo-cho, Sayo-gun, Hyogo, Japan; 2 Graduate School of Science, Technology and Innovation, Kobe University, Kobe, Hyogo, Japan; 3 Graduate School of Medicine, Kyoto University, Kyoto, Kyoto, Japan

## Abstract

Ras proteins are small GTPases that regulate cell proliferation, migration, and survival by cycling GTP-bound and GDP-bound forms with conformation change at two loop regions. The GTP-bound form is in equilibrium between closed state 2 which is able to bind with downstream effectors, and open state 1 which is not able to. Hydrolysis of GTP to GDP changes structure of switch regions into GDP-bound form, which disrupt effector binding and inactivate signaling. However previous time-resolved crystallographic studies of Ras protein focused on the inactivation process, they did not illustrate overall of this process because of crystal packing. Then, we aimed to visualize it by X-ray crystal structure analysis with new space group.

For crystal structure analysis, we prepared H-Ras protein bound to NPE-caged GTP, which generates GTP upon UV light irradiation, and explored crystallization conditions for microcrystal. The microcrystals suspension were irradiated with UV light to generate GTP in the crystal. After various times of incubation, they were mounted on 1 mm loops, and frozen with liquid nitrogen to trap states. We collected serial diffraction data by SS-ROX method [1], raster-scanning with rotation, at the SPring-8 synchrotron radiation facility beamline BL41XU, performed data processing, and determined the crystal structure.

Ras protein bound to caged-GTP was in the open State 1 structure due to steric hindrance of the cage moiety. This state 1 was maintained in the crystal even after removing the cage by UV light irradiation, although with slight structural changes. As a result of extending the incubation time before freeze-trapping from 15 min to 20 hr, difference electron density indicating the progression of hydrolysis of γ-phosphate of GTP generated by photoirradiation was observed. Electron density for any nucleophilic water candidates previously reported in state 2 [2] were not observed in the series of freeze-trap data. Structural change in the switch I region was also observed. The newly appeared electron density matched the model of the switch region of the GDP-bound form. We explain how this conformational transition is achieved in this crystal packing, by comparing with previous studies of time-resolved Ras crystal structure analysis.
